# Mother-child interactions and young child behavior during procedural conscious sedation

**DOI:** 10.1186/s12887-016-0743-2

**Published:** 2016-12-03

**Authors:** Daniella Miranda-Remijo, Mara Rúbia Orsini, Patrícia Corrêa-Faria, Luciane Rezende Costa

**Affiliations:** 1 Health Sciences Graduate Program, Faculdade de Medicina, Universidade Federal de Goiás (UFG), Rua 235, Setor Universitário, CEP 74605-020 Goiânia, Goiás Brazil; 2 Faculty of Psychology, Faculdade de Educação, Universidade Federal de Goiás, Rua 235, Setor Universitário, CEP 74605-050 Goiânia, Goiás Brazil; 3 Dentistry Graduate Program, Faculdade de Odontologia, Universidade Federal de Goiás, Primeira Avenida, Setor Universitário, CEP 74605-220 Goiânia, Goiás Brazil; 4 Faculty of Dentistry, Faculdade de Odontologia, Universidade Federal de Goiás, Primeira Avenida, Setor Universitário, CEP 74605-220 Goiânia, Goiás Brazil

**Keywords:** Parent-child relations, Parenting, Child-rearing, Child behavior, Conscious sedation

## Abstract

**Background:**

As many preschoolers are not able to cooperate with health-related invasive procedures, sedation can help with the child’s comfort and allow the intervention to be done. It is scarcely known how parents affect children’s behavior during dental treatment under conscious sedation. The aim of this exploratory study was to analyze the association between mother-child interactions in day-to-day family life and preschool children’s behavior during dental treatment under conscious sedation.

**Methods:**

This cross-sectional study included 27 children aged 2–6 years and their mothers. The children’s behavior during dental treatment under conscious sedation was verified through the analysis of videos and using an observational scale. Social skills of mothers were verified through interviews using the Parental Educative Social Skills Interview Script (RE-HSE-P); the sum of the scores allowed the establishment of the categories “clinical” and “non-clinical”. We presented descriptive analyses and bivariate associations.

**Results:**

Children’s overall behavior during dental sedation was: very poor (*n* = 2), poor (*n* = 1), regular (*n* = 2), good (*n* = 9), very good (*n* = 9) and excellent (*n* = 4). Social skills varied: parental educational social skills (*n* = 24 clinical vs. *n* = 3 non-clinical); child social skills (*n* = 20 vs. *n* = 7), context variables (*n* = 15 vs. *n* = 12), negative educational practices (*n* = 12 vs. *n* = 15), child behavior problems (*n* = 7 vs. *n* = 20). There was no association between child behavior under sedation and social skills categories (*P* > 0.05).

**Conclusions:**

The majority of interviewed mothers reported issues in parental educational social skills and child social skills, which did not affect the outcomes of the children’s behavior during the procedural conscious sedation.

## Background

The parenting concept has been understood as two constructs that exist simultaneously but can be assessed separately: the values and goals of a parent as a caregiver, as well as the parental skills and strategies to accomplish their goals [1]. Parenting behaviors affect children’s health and social outcomes [1]. For example, satisfactory general behavior in children is associated with parents’ positive educational practices and children’s favorable social skills [2]. Also, there is evidence that parental factors such as more inter-parental conflict, aversiveness, less warmth, more abusive parents and over-involvement are related to depression and internalizing of problems in children but not to anxiety [3, 4]. As the relationship between parenting and child mental health has significant impacts later in life, programs focusing on improving parenting skills have been proposed, applied and investigated [5].

At preschool age, children can show a distress behavior in response to health procedures – immunization, venipuncture, lumbar puncture, diagnostic imaging tests, outpatient minor surgery, perioperative procedures, dental treatment, etc. Their challenging behavior during such procedures can be related to several factors, including parenting behaviors. In the anticipatory phase of an immunization, for example, children showing resistance and behavioral distress are related to adults displaying low activity and/or less efficient support behaviors [6].

Dental fear and anxiety and dental behavior management problems occur in 9% of children and adolescents and have been related to several psychological factors [7]. Nevertheless, the association between parenting styles and children’s behavior during a dental procedure has scarcely been investigated and the results are controversial. Whilst no relation was found between parenting style and children’s anxiety and behavior during dental treatment [8, 9], one study reported that authoritative parenting is related to less caries and better behavior during the first dental visit, whilst permissive parenting associates with worse behavior and increased caries [10]. Moreover, in a pilot study, the child’s behavior was related to maternal emotional intelligence which, in turn, was associated with authoritative parenting style [11]. This latest research team confirmed the correlation between permissive parenting style and behavior problems and anxiety in children during dental treatment [12].

Many preschool children with early childhood caries (ECC) cannot cooperate with the dental treatment; so they are referred for procedural conscious sedation. In fact, there is a relation between children’s challenging dental behavior requiring sedation and permissive parenting style [13]. Conscious sedation can be a solution to manage children’s behavior during a medical/dental procedure, but the success of different sedative regimens depends on several factors related to the child, the health care team and the context. However, there is a lack of knowledge on how parenting affects children’s behavior during procedural sedation. In this exploratory study, we aimed to understand the association between mother-child interactions in day-to-day family life and preschool children’s behavior during dental treatment under conscious sedation.

## Methods

### Ethical procedures

This study was approved by the Institutional Research Ethics Board of the Federal University of Goias; written informed consent was obtained from the parents of each child, and all the procedures followed were in accordance with Resolution 466/2012 of the National Health Council, Brazil, and the Helsinki Declaration.

### Study design, participants and setting

This cross-sectional study, nested in two randomized clinical trials (NCT02284204 and NCT02447289), was performed with mother-child dyads. To be included in those trials, children had to be healthy, 2–6 years old, requiring dental restorative procedures but with history of negative behavior during previous dental appointments. To be included in this specific study, mothers had to be literate.

Outpatient procedural sedation was performed in the UFG dental sedation clinic (“Núcleo de Estudos em Sedação Odontológica” – NESO), following guidelines for efficient and safe sedative protocols [14].

### Sedation and clinical procedures

An anesthesiologist and/or a pediatrician were in charge of the sedation procedures. The sedation was performed according to the studies’ protocols: midazolam only or a mixture of ketamine and midazolam. Midazolam was supplied as an oral solution containing 2.0 mg/mL of midazolam maleate (Dormire®, Cristália, São Paulo, Brazil). Ketamine was supplied as a parenteral formulation containing 50 mg/mL of ketamine hydrochloride (Ketamim S®, Cristália, São Paulo, Brazil). Midazolam and ketamine were dosed and mixed in a syringe just before their administration to a child. The physician administered the sedative with a syringe after confirming the child’s health condition and a fasting period of 6 h.

The children underwent restorative treatment under local anesthesia (2% lidocaine with 1:100,000 epinephrine) and operative field isolation. The restorative dental treatment was performed by one pediatric dentist, supported by a dental assistant and a health professional continuously monitoring the children’s vital signs (oxygen saturation and heart rate) and behavior. Mothers sat down in the dental chair with their children throughout the appointment and were allowed to comfort them verbally and/or help in their physical restraint when necessary.

### Children’s behavior assessment

Each dental sedation appointment was fully filmed by a digital camera positioned out of the children’s reach. Then, the recorded digital files were watched extensively by five trained and calibrated dental students (weighted Kappa > 0.6), who registered the children’s behavior every minute from the beginning until the end of the procedure, using the Houpt scale [15]. The Houpt scale is designated to assess children’s behavior during pediatric dental sedation. It comprises the following categories/descriptions: (A) Sleep – (1) awake; (2) drowsy, disoriented; (3) sleeping; (B) Movement – (1) violent, (2) continuous, (3) intermittent, (4) absent; (C) Crying – (1) hysterical, (2) continuous, (3) intermittent, (4) absent; Overall behavior – (1) very poor, treatment aborted, (2) poor, (3) regular, (4) good, (5) very good, (6) excellent. Relative frequencies for each score throughout a session were calculated.

### Mother-child interactions (parental educative social skills)

The parental educative social skills were evaluated through the interviews with mothers previously to the dental sedation appointment, in the UFG Qualitative Laboratory at the Dental School. One trained psychologist interviewed the mothers, using the Brazilian validated instrument “Roteiro de Entrevista de Habilidades Sociais Educativas Parentais” (RE-HSE-P; translation by the authors of the instrument: Parental Educative Social Skills Interview Script). The development of the RE-HSE-P was reported in 2010 [16]. The instrument is based on a semi-structured clinical interview and consists of 13 main questions followed by secondary inquiries. For evaluation, data is organized into three broad categories: communication, expression of feelings/coping and setting limits [16]. These categories allow the assessment of the mother-child interaction. The category “communication” is related to the parents’ ability to talk and question. “Expression of feelings/coping” is related to the ability to express feelings, opinions, show affection, and play, while the category “setting limits” involves the act of identifying and evaluating the result of socially skilled and unskilled behavior, establish rules, keeping promises, identify errors and apologize [16]. The RE-HSE-P evaluates positive and negative practices of education, context, behavior problems and children’s social skills [17]. According to the instruction of the instrument, the sum of the scores allows the establishment of the categories “clinical”, “non-clinical” and “borderline”. These categories guide the referral for psychological intervention: clinical – requires psychological intervention to promote greater emotional adjustment and more effective adaptation; non-clinical – does not require psychological intervention; and borderline – features a narrow margin from clinical and may also be referred to psychological intervention or just education. For bivariate analysis, the borderline category was classified as clinical in this study. The interviews were audiotaped and transcribed verbatim for analysis by the same psychologist. During the interviews, mothers were also asked about socioeconomic indicators.

### Pilot study and sample size estimation

A pilot study involving five children and mothers was carried out to test the methods. The results of this pilot study indicated there was no need to change the proposed methods. Calculation of the sample size was carried out considering the findings obtained in the pilot study. The variables “parental educational social skills” (clinical/non-clinical) were tested with child’s favorable or unfavorable behavior during dental sedation. In the clinical group, 50% of children had favorable behavior, whilst, in the non-clinical group, 100% of children showed favorable behavior. Taking into account a 95% confidence interval and 5% standard error, the minimum sample size was determined to be 11 children in each group.

### Statistical analysis

We entered data in the Statistical Package for Social Sciences (SPSS for Windows, version 19.0, SPSS Inc., Chicago, USA) and analyzed it considering the variables: dependent – child’s behavior; independent – child’s demographics (age, sex, school attendance), maternal demographics (age, formal education, family income) and social skills. Statistical analysis involved the Shapiro-Wilk test, descriptive analyses and bivariate associations (Mann-Whitney, chi-square test) considering the significance level of 5%.

## Results

The respondents included were 27 mothers, with a mean age of 32.0 years (22 to 45 years) with variable educational backgrounds: primary – incomplete (*n* = 3) or complete (*n* = 2); secondary – incomplete (*n* = 3) or complete (*n* = 15); tertiary – incomplete (*n* = 1) or complete (*n* = 2); one mother did not answer. Most of them were married (*n* = 15) or in a stable relationship (*n* = 9); the others were single (*n* = 2) or divorced (*n* = 1). Thirteen mothers had a job and worked for one (*n* = 3) or two periods (*n* = 10). They reported a monthly family income (Brazilian minimal wage “BMW” – approximately U$207) of: ≤1 BMW (*n* = 8), 2 BMW (*n* = 9), 3 BMW (*n* = 9) and 4 BMW (*n* = 1).

A total of 27 children (17 boys and 10 girls) aged 23 to 83 months (mean 54.4 months) underwent dental treatment under sedation and had their behavior assessed through digital video files. Nine were the only children and the others had 1 (*n* = 8), 2 (*n* = 7), 3 (*n* = 2) or 4 (*n* = 1) siblings. The majority (*n* = 17) attended school.

Two children received midazolam only and the other 25 received the mixture of ketamine plus midazolam. The children’s overall behavior during dental sedation was frequently satisfactory and comprised the six Houpt scale categories: aborted (*n* = 2), poor (*n* = 1), regular (*n* = 2), good (*n* = 9), very good (*n* = 9) and excellent (*n* = 4). For the purpose of bivariate analysis, those six categories were grouped into two: more favorable behavior (Houpt good to excellent, *n* = 22) and less favorable behavior (Houpt aborted to regular, *n* = 5).

Based on the observed scores in the RE-HSE-P, respondents were grouped into clinical and non-clinical groups (Table 1). The majority of mothers were problematic regarding parental educative social skills but reached non-clinical total positive scores, whilst the majority of children were categorized as clinical for their social skills although only seven were reported as having clinical behavior problems. There was a balance between clinical and non-clinical groups regarding context variables, negative educational practices and total negative scores.

**Table 1:** Association between children’s behavior during procedural dental sedation and clinical groups based on family social skills

	n	More favorable behavior (*n* = 22)	Less favorable behavior (*n* = 5)	*P* ^a^	Effect size (Phi)
Parental educational social skills	24			0.25	0.17
Non clinical		3 (100.0%)	0 (0.0%)		
Clinical		19 (79.2%)	5 (20.8%)		
Child social skills	20			0.07	0.37
Non clinical		4 (57.1%)	3 (42.9%)		
Clinical		18 (90.0%)	2 (10.0%)		
Context variables	15			0.07	0.34
Non clinical		8 (66.7%)	4 (33.3%)		
Clinical		14 (93.3%)	1 (6.7%)		
Negative educational practices	12			0.07	0.34
Non clinical		14 (93.3%)	1 (6.7%)		
Clinical		8 (66.7%)	4 (33.3%)		
Child behavior problems	7			0.07	0.28
Non clinical		15 (75.0%)	5 (25.0%)		
Clinical		7 (100.0%)	0 (0.0%)		
Total positive scores	7			0.07	0.28
Non clinical		15 (75.0%)	5 (25.0%)		
Clinical		7 (100.0%)	0 (0.0%)		
Total negative scores	14			0.15	0.27
Non clinical		12 (92.3%)	1 (7.7%)		
Clinical		10 (71.4%)	4 (28.6%)		

^a^Likelihood ratio chi-square

In the first bivariate analysis strategy, we investigated the relationship between the frequencies of the RE-HSE-P categories for the non-clinical/clinical groups and children’s behavior (Table 1). Most children from the non-clinical group had better behavior during the dental sedation care, while the majority of those included in the clinical group had negative behavior (no statistically significant associations; effect size values (Phi) small to medium).

Then, we ran another bivariate analysis, considering the scores of the mothers’ responses to the RE-HSE-P and the other variables collected (Table 2). The child’s behavior groups did not differ regarding social skills scores, except for context variables and total positive scores, where children with less favorable behavior reached higher scores than those with more favorable behavior (small effect size).

**Table 2:** Children’s behavior during procedural dental sedation related to family social skills and other variables

Variables	Median (interquartile range) or n (%)		*P*	Effect size
	More favorable behavior (*n* = 22)	Less favorable behavior (*n* = 5)		
*Children’s characteristics*				
Age (months)	55.0 (19.0)	51.0 (31.0)	0.88^a^	0.03
Boys	15 (68.2%)	2 (40.0%)	0.25^b^	0.23
Attends school	13 (59.1%)	4 (80.0%)	0.36^b^	0.17
*Family aspects*				
Number of siblings	1.0 (2.0)	1.0 (1.0)	0.21^a^	0.24
Family income	2.0 (2.0)	3.0 (2.0)	0.08^a^	0.34
*Maternal characteristics*				
Age	32.0 (12.0)	30.0 (19.0)	0.98^a^	0.01
Completed primary education	17 (81.0%)	4 (80.0%)	0.96^b^	0.01
Has a job	9 (40.9%)	4 (80.0%)	0.11^b^	0.30
*Social skills (score)*				
Maternal educational social skills	6.5 (3)	7.0 (2)	0.65^a^	0.09
Child social skills	8.0 (2)	11.0 (5)	0.15^a^	0.05
Context variables	8.5 (4)	11.0 (6)	0.03^a^	0.12
Negative educational practices	5.0 (4)	8.0 (6)	0.11^a^	0.23
Child behavior problems	6.0 (7)	7.0 (3)	0.93^a^	0.01
Total positive scores	26.0 (6.0)	30.0 (12.0)	0.03^a^	0.05
Total negative scores	12.5 (9)	18.0 (10.0)	0.34^a^	0.16

^a^Mann Whitney test

^b^Likelihood ratio chi-square

## Discussion

This study with a group of mothers of children referred to conscious dental sedation because of behavior management problems shows that the sedative protocol was able to disrupt the expected mother-child interaction in the dentist’s chair. That is, children who showed worse behavior during sedation had mothers with higher scores of positive parenting practices. These results are provocative and should be discussed from the perspective that this is an exploratory study, and generalizations are not advisable.

The participants of this study have extreme characteristics regarding the main variables investigated, i.e., parental educational social skills and child dental behavior. Most mothers had a low parental educational repertoire of social skills. In addition, all children evaluated in this study were referred for dental treatment under sedation because they did not cooperate with previous attempts to provide dental treatment. That is, they moved and cried a lot during such attempts, and the realization of planned procedures was not possible. By the way, the sedation worked quite well for the majority of children included in the analyses.

In this study, we used a validated instrument (RE-HSE-P) that investigates parental educative social skills. The positive parental practices in the RE-HSE-P are positively related to favorable indexes in parenting style [18]. Therefore, it is legitimate to compare our results with other investigations of parenting styles and practices.

We were not able to show a significant association between parental educational styles and behavior of sedated children. This is contrary to our initial expectation that parental educational style, reported by the mother, still has some interference in the behavior of the sedated child, because it is conscious sedation, i.e., there is some interaction between the child and the dentist despite the sedative. In addition, the mother sat with the child in the dental chair during the entire procedure.

Still, one of the sedatives used was ketamine, very well known for provoking a dissociative anesthesia, “a form of anesthesia characterized by catalepsy, catatonia, analgesia and amnesia” [19]. As we used a sedative dosage for ketamine, and not an anesthetic one, we could expect interference in the transmission of the sensory signals to the cerebral cortex, as well as in the reaction against those signals [19]. In this study, only two children received midazolam as the sole sedative drug; the other 25 received the association ketamine/midazolam. So, it was not possible to compare the two sedative regimes regarding their association with children’s behavior and parental educational social skills. Another investigation showed the superiority of midazolam/ketamine for children’s behavior management when compared with midazolam alone, although less cooperative behaviors could also be observed in children that received ketamine [20]. In fact, the body of evidence points out to the efficacy and safety of ketamine in pediatric procedural sedation [21]. Thus, one can hypothesize that children who receive ketamine have less interference of parental styles because of the dissociative effect of the drug, but this has to be confirmed in studies with more homogeneous distribution of sedative regimes among groups.

In addition, the small sample size can explain the lack of association between parental educational social skills and behavior of sedated children. There was a small number of children with negative behaviour (only five). Considering the initial hypothesis of the association between negative behavior and clinical social skills, the sample size was not sufficient to detect differences among children with more or less favorable behavior according to the clinical or non-clinical parenting social skills.

However, there was a trend of association between some problems in reported social skills and children’s behavior, which could be confirmed in larger samples. Problems with child social skills, context variables, child general behavior and total positive scores were more frequent in the group comprising children with more favorable behavior. This is intriguing and might be explained by the low number of children categorized as presenting less favorable behavior and by the effect of the sedative, which would mediate the child-mother interaction during a procedural sedation. Nevertheless, our results agree with other reports on non-sedated children, which observed more variability in social skills in families of children without general behavior problems, compared with children with general behavior problems [2].

On the other side, problems with negative educational practices reported by mothers were more observed in the group with less favorable behavior, which makes sense. According to a study conducted in Iran, children of authoritarian parents present worse behavior during a dental appointment (without sedation), compared with those of authoritative parents [13]. Also, fewer externalizing behaviors in children are related to greater levels of skill encouragement by parents [22]. Interestingly, in a study on parents of Latino children living in the US, higher levels of externalizing behaviors in children were predicted by fathers’ reports of non-coercive discipline and problem solving [22]. In this study, we did not investigate the fathers’ perspective, but one of the items of the RE-HSE-P is about parental agreement in child education (question 8), which did not differ regarding the comparison groups.

Another relevant finding in this study was that, beside the large number of mothers with clinical parental educational social skills, children had low social skills, pointing to a characteristic of children with resistance to dental care. These results for children’s social skills make sense, because there is need for the child to develop a social skills repertoire to face and deal with challenges [23].

One cannot deny the role of child temperament as a mediator in the effect of parenting [24]. Children with more difficult/unadaptable temperaments respond more negatively to a lack of positive parenting and, then, present worsened externalizing and internalizing behavioral problems, compared with those without such temperamental characteristics [24]. Future studies should investigate the association among parental educational social skills (or parenting styles), child behavior during procedural sedation and child temperament.

The results of this study, despite the limitations in the statistical significance, show families in need of social skills but, on the other hand, a beneficial effect of conscious sedation to reduce the suffering of the preschool child in the dentist’s chair, as already shown [20]. Thus, parenting practices should also be addressed by a multidisciplinary health care team for the child in need of elective procedures under pharmacological intervention. Future studies should investigate whether such an approach favors the effectiveness of sedation for this population.

This study, although exploratory in nature, has some weaknesses such as the small sample size, which should be minimized by the very controlled group of participants’ conditions and procedures. Also, in the data analyses, we observed that the majority of children presented favorable behavior, which made it difficult to identify an association between parental educational social skills and less favorable behavior. This favorable behavior was due to the conscious sedation that was able to control the negative behavior children had in previous consultations; other investigations should compare children with negative and positive behaviors during procedures with regard to parental educational social skills. These issues should be addressed in future studies.

Altogether, this exploratory investigation provides an original and relevant insight towards parental educational social skills and child behavior during a procedural sedation and should encourage: 1. Greater interaction between psychologists and physicians/dentists in providing pharmacological intervention for the pediatric population; and 2. The parental presence during procedural sedation in children.

## Conclusions

The majority of interviewed mothers reported issues in parental educational social skills and child social skills, which did not affect the outcomes of the children’s behavior during the procedural conscious sedation.

## Abbreviations

BMW: Brazilian minimal wage
ECC: Early childhood caries
NESO: Núcleo de Estudos em Sedação Odontológica
RE-HSE-P: Roteiro de Entrevista de Habilidades Sociais Educativas Parentais
SPSS: Statistical package for social sciences
UFG: Universidade Federal de Goiás
